# Ghanaian women’s experiences of unsuccessful in-vitro fertilisation treatment, unravelling their meanings: a Heideggerian hermeneutic phenomenological study

**DOI:** 10.1186/s12884-024-06365-7

**Published:** 2024-03-20

**Authors:** Amoah Vida Maame Kissiwaa, Nicola Fouché

**Affiliations:** 1 School of Nursing and Midwifery, Kwame Nkrumah University of Science and Technology (KNUST), Kumasi, Ghana; 2 Department of Health and Rehabilitation Sciences, Division of Nursing and Midwifery, University of Cape Town, Cape Town, South Africa

**Keywords:** Experiences, Heidegger’s Hermeneutic Phenomenology, Infertility, Meaning, Unsuccessful IVF treatment

## Abstract

**Background:**

Women having experienced infertility over a period usually decide on an option for an invitro fertilisation treatment (IVF). However, in the quest to seek help and to be part of motherhood, they sometimes become unsuccessful in their fertility journey. The researchers aimed to explore the meanings and emotions attached to infertility and unsuccessful invitro fertilisation (IVF) treatment among Ghanaian women, as this area of inquiry is less explored in Africa and specifically in the Ghanaian context.

**Methods:**

The study followed a qualitative approach and drew inspiration from the Heideggerian phenomenological philosophy and design. Six (6) women, aged between 29 and 40 years, who had experienced at least one unsuccessful IVF cycle, were purposefully selected from a private specialist fertility hospital in Kumasi, Ghana. One-on-one interviews were conducted with the participants, and the interviews were transcribed verbatim. The collected data was analyzed using Van Manen six-step framework, which helped to uncover the existential meanings and interpretations these women ascribed to their experiences.

**Results:**

The results of the study revealed four main themes that were deemed essential aspects of meaning for the participants. These themes were: (1) Experiencing an Existential Faith and Hope. The participants described their journey through infertility and invitro fertilisation (IVF) treatment as a profound test of their existential faith and hope finding strength in maintaining a positive outlook despite the challenges and setbacks they faced., (2) Facing up to the *Angst*: This theme highlights the participants' courage in confronting the emotional dimensions of their struggles. The women acknowledged and confronted their anxieties, fears, and emotional distress associated with their infertility and unsuccessful IVF treatment.*,* (3). Non-disclosedness: This theme refers to the participants' experiences of keeping their struggles with IVF treatment private, often due to societal and cultural factors. (4). Endured feelings of inadequacy of *being-in-the-world-of-motherless*: Participants expressed feelings of inadequacy, self-doubt, and a sense of being incomplete due to their inability to conceive and fulfill the societal role of motherhood. Their narratives revealed the profound impact of societal expectations on their self-perception and identity.

**Conclusion:**

The study's findings reveal insights into the experiences and interpretations of infertility and unsuccessful IVF treatment among Ghanaian women. Employing Heideggerian hermeneutics, the research elucidates the diverse existential, emotional, and societal aspects inherent in the struggles of infertility. The multifaceted nature of these women's journeys underscores the significance of a comprehensive approach to infertility care that recognizes the cultural, social, emotional, and existential dimensions of the IVF process. Additionally, the study emphasizes the necessity for culturally sensitive support systems and interventions to address the unique challenges faced by this population.

## Background

Globally, infertility is perceived as a reproductive health issue, where more than 15% of the world’s population report that there is an issue of becoming pregnant naturally [1, 2]. Researchers have described infertility as a sexually active couple not being able to fall pregnant after one year of regular sex without using any protection [3, 4].

Being infertile is a challenging part of a woman's *lifeworld* and for some decades identified with women in low-resourced areas in Africa [5]. Fertility is highly regarded in many cultures within Ghanaian communities and, having children is among the most essential purpose of life-giving for women generally. Though both male and females are affected by infertility as a reproductive health problem, some recent research outcome have revealed that the focus on women is more apparent when compared to men. Women who suffer from infertility may endure considerable societal humiliation in many African cultures [6, 7].

Invitro fertilisation treatments has become a powerful medical intervention in helping millions of people achieve their hopes of having a baby. For some women, treatment will not be successful, leaving many to cope with unresolved infertility [8, 9]. The positive results of IVF treatment have been steadily increasing over recent years. Studies conducted in the United States showed that the overall clinical pregnancy rate was 40% per treatment cycle from 2017 to 2019 [10, 11]. This means that 60% of women are unable to conceive successfully with each treatment cycle and 20% of women will fail to conceive after three attempts following IVF treatments [12]. Such unsuccessful IVF treatments have a negative effect on the mental and social well-being of the affected women [9, 13].

The question posed: ‘what are the lived experiences of women experiencing a single or repeated failure of embryo transfer following IVF [12] and the common meanings assigned to this unique experience?’ remains unanswered in Ghana and certain parts of Africa.

Specifically in the literature search, limited studies have been found in Africa and none in Ghana that were specific to uncover the lived experiences of women following unsuccessful IVF treatment in the Ghanaian context. Furthermore, research on the distinctive meanings and detailed understanding of this intricate phenomenon in sub-Saharan Africa is inadequate [14]. There is therefore a wide knowledge gap in literature on the subject under study. It is envisaged that the findings of the study may assist in developing indigenous, culturally relevant practical interventions to guide effective nursing practices in assisted reproductive technology/invitro fertilisation treatment (ART/IVF) services and support systems to help reduce the impact on their quality of life.

## Aim of the study

The study sought to uncover the profound *meanings* and emotions women struggling with infertility attributed to their experiences of unsuccessful invitro fertilisation treatment.

### Heidegger and *meaning*

*Meaning* (*Sinn*) as explained by Heidegger is an understanding that stems from a projection and enables us to understand a particular *entity*^1^ wherein something maintains itself intelligibility^2^ [15]. Heidegger argued that individuals act on the basis of the meaning that a particular entity has for them, derived from the personal experiences of people, intertwined with their social and cultural interactions, and adapted by interpretations [16, 17].

The main theme underpinning the philosophy of Heidegger is the question related to the *meaning* of *Dasein’s being* [18, 19]*.* For Heidegger, his attention to hermeneutic research is focused on the individual *life-world* (*Lebenswelt*) rather than revealing knowledge through consciousness [20]. He developed phenomenology as a hermeneutic or interpretive philosophy, with the aim of uncovering the *meanings* in people’s life experiences [16, 17, 21].

Heidegger believes that lived experience gives *meaning* to each person’s perception of a phenomenon [22]. As emphasised by van Manen the *meaning* of hermeneutic phenomenological philosophy ultimately lies in interpretation [23]. Through phenomenological conversations, the researcher strives to gain entrance into the participant’s world to uncover the *meaning* of participants’ lived experiences.

## Methods

### Study design

Heidegger’s hermeneutic phenomenology was utilized to unravel the *meanings* women attribute to their life when undergoing IVF treatment and following unsuccessful IVF procedures. Heideggerian hermeneutics phenomenology sought to interpret the human lived experiences using language to provide both understanding and knowledge [24, 25]. In order to respond to the main research question: *"What does it mean to be motherless-in-the-world-of-motherhood and experiencing IVF treatment failure?"* Heidegger's Hermeneutic philosophy of phenomenology was deemed an appropriate philosophical stance.”

### Study site

The site of the research was Ruma Fertility and Specialist Hospital located in Kumasi, in the Ashanti region. Ruma Fertility Hospital in Ghana is a cutting-edge specialist hospital primarily focused on providing first-rate infertility treatment services in Kumasi and beyond.

### Invitation of the participants, sampling and sample size

Following, ethics approval, from the Faculty of Health Sciences Human Research and Ethics Committee (HREC) and all the research committees at the Fertility and specialist hospital, participants were identified and invited from the fertility’s clinic database if they met the inclusion criteria for this study. A purposive sampling method was used to invite six women who resided in the Kumasi metropolis, who were not successful following at least a cycle of IVF treatment prior to the initiation of the study.

### Expression of rigor

To ensure the credibility of this study, we adhered to the criteria of rigor outlined by de Witt, Ploeg, and van Manen in hermeneutical phenomenological research, encompassing balanced integration, openness, concreteness, resonance, and actualization [23, 26]. De Witt and Ploeg [26] introduced 'balanced integration' as the first aspect of rigor, encompassing three essential elements: the articulation of philosophical principles, a well-defined methodological approach, and ensuring a balanced representation of participants' voices. In this approach, the study establishes a solid foundation by delivering clear and well-articulated philosophical concepts that seamlessly connect with the phenomenon under investigation. The study's philosophical underpinning aligns with Heidegger's hermeneutic philosophy, specifically focusing on the three modes of being: *authenticity*, *inauthenticity*, and *undifferentiatedness*. This alignment is deemed appropriate and consistent with the research objective, contributing to the overall coherence of the study. Through the incorporation of participants' voices and a philosophical method, the study gives expression to the lived experiences of the individuals involved."

Openness and balanced integration were maintained throughout the research process, allowing scrutiny and harmonizing the phenomenon of interest. Concreteness was emphasized, ensuring clear writing and providing concrete examples to authenticate the study's context. Trustworthiness was reinforced through rich descriptions, quotations, member checking, prolonged engagement, peer review, and debriefing. Resonance aimed to integrate meaning into study findings, fostering a profound understanding for the reader. Actualization considered the future implications of participants' phenomenological interpretation, aligning appropriately with the study findings. The study effectively gives voice to the lived experiences of women through participant narratives and a philosophical approach.

### Data collection process and procedure

Information was gathered through phenomenological dialogues that lasted between 40 and 90 min. Before the phenomenological conversation began, permission was obtained from the participants to tape record the conversation for the duration of the information generation process, to which they readily agreed. During the conversation a semi-structured phenomenological conversational prompt was used which consisted of keywords and prompts to help the researchers stay focus on the phenomenological conversations.

In total, two different phenomenological conversations with two follow-up sessions with each participant were carried out over a 24-month period. Prior to the first phenomenological conversation, the primary researcher engaged in an open-ended narrative conversation with two women with infertility problems. This assisted in preparing for the actual phenomenological conversation in various ways, including learning how to use the tape recorder, practicing silence, and dealing with interruptions.

The researchers' long-term involvement enabled them to re-examine crucial concerns and ponder on new areas of concern. The researchers gained comprehensive and information-rich material from the participants' lived-world through the two phenomenological conversations. This long-term involvement seems to increase the researchers' and participants' trusting connection [27].

All phenomenological conversations and feedback sessions were originally conducted in the Twi language and subsequently transcribed verbatim into English. To extract conceptually relevant information, a series of translation techniques were employed. Initially, the content from phenomenological conversations and observational documents was transcribed verbatim in Twi. Subsequently, these Twi transcripts were translated into English. To ensure translation accuracy, an independent bilingual individual was tasked with translating the English version back into Twi. A comparison between these two versions was then conducted to verify the accuracy of the translation in relation to the original transcripts. The goal was to capture the similarities in the phenomenological conversations. The final English-translated conversations were utilized to identify emerging themes from the participants' narratives, thus informing the hermeneutic interpretive process."

### Unravelling of the phenomenological conversations/data analysis

For this study, the researchers followed van Manen’s (1990) “six research activities” [23] which briefly are:

1. Turning to the nature of the lived experience: To be able to achieve in-depth *meaning* from the women’s shared lived experiences, we engaged with their narratives by highlighting sections of the transcribed phenomenological conversations and reflected on these through a personal journal. In particular paying attention to their tone of voice and physical gestures such as facial expressions, periods of silence and even them being tearful so that the *essence* of *meaning* in their *lived-world* was captured.
2. Investigating experience as we live it rather than as we conceptualize it: Information was gathered not only through the participants’ phenomenological conversations, but also through writing, and observation. In staying true to Heidegger’s philosophical tenets of *Dasein, being-in-the-world,* the three *existential* modes of *being* (*authenticity, inauthenticity,* and *undifferentiatedness*), we started to identify the naïve themes of the participants’ experiences.
3. Reflecting on the essential themes which characterize the phenomenon: As suggested by van Manen, themes can be isolated from the participant’s descriptions of experience by three different approaches which include: 1. The wholistic or sententious reading approach. 2. The selective or highlighting reading approach and 3. The detailed or line-by-line reading approach. To grasp the essential *meaning* of their experiences under study, these three approaches were applied at different times and to different narratives during the unravelling of the phenomenological conversations.
4. Describing the phenomenon through the art of writing and rewriting: Through the process of writing and rewriting of the phenomenological conversations, *meaning* is revealed. Using a digital audio recorder, the phenomenological conversations were captured and transcribed verbatim as soon as possible.
5. Maintaining a strong and orientated relation to the phenomenon: To be oriented in the study, the researchers made every effort to stay focused when analyzing the information by continually referring back to the research question to avoid ‘trivialities and falsities’ as described by van Manen [23]. The lived experience themes identified were then used during the interpretation stage to assist in the selection of the relevant quotes from the participant’s transcribed phenomenological narratives.
6. Balancing the research context by considering parts and whole: In this final stage of the research process, van Manen advocates that there is the possibility of the researcher losing sight of the phenomenon being studied and can get stuck in the information consequently losing its *meaning*. Heidegger asserts that to comprehend the meaning of a conversational text, one must understand the meaning of its parts. However, understanding these parts is only possible through anticipating the overall meaning of the text [22]. Every effort was made to balance the research context by considering parts and whole by continually scrutinizing the participant’s life-world and moving between parts of the phenomenological conversational text with that of the experience being shared. The interpretive analysis and participant feedback were completed when theme saturation was achieved, and no new lived experience themes emerged. As a result, the conversations were considered comprehensive, offering rich insights into the phenomenon.

## Results

A total of six women were invited and participated in the study. The age of the women in the study ranged between 29 to 40 years at the time of the study. The average age of the participants was 30 years old. All of the participants were married and all gainfully employed. Twi (Ghanaian Akan Language) was the first language for majority of the participants. Three participants had undergone IVF treatment for three consecutive times. Mostly, the cause of infertility was tubal blockage (Table 1).

**Table 1:** Socio-demographic characteristics of study participants

Variables	Frequency (*n* = 6)	Percent (%)
**Age**		
20–30 31–40	4 2	66.7 33.3
**Educational level**		
Primary Tertiary	2 4	33.3 66.7
**Religion**		
Christian Muslim	5 1	83.3 16.7
**Occupation**		
Banker Insurance broker Nurse Teacher Trader	1 1 1 1 2	16.7 16.7 16.7 16.7 33.2
**Infertility duration**		
2–7 yrs 8–13 yrs	4 2	66.7 33.3
**Causes of infertility**		
Polycystic ovarian syndrome (pcos) Tubal blockage Unknown	1 4 1	16.7 66.6 16.7

## Emerging themes

Utilizing van Manen’s six research steps to unravel the experiences of the women’s narratives of unsuccessful IVF treatment and keeping to Heidegger’s phenomenon, [23] the study identified four themes as being essential aspects of *Meaning* attributed to Ghanaian women undergoing IVF treatment. The themes reflect the shared meaning of IVF treatment experiences of the women in this study. The major themes and the subthemes that emerged from the participant’s phenomenological conversations and unraveling of the transcripts are presented in Table 2.

**Table 2:** Lived experience themes

**Heidegger’s mode of *being***	**Lived experiences’ phenomenological themes**
***Authenticity*** ***(mine-self)***	**Experiencing an existential faith and hope** • A feeling of an *existential* faith • A sense of optimism to celebrate a successful motherhood
***Inauthenticity*** ***(they-self)***	**Facing up to the *angst* (*angst,* [*verfallen*] *fallenness*)** • *Angst* of uncertainty • Unpleasant nature of emotional drain • Causing huge financial losses • Sense of guilt and self-blame **Non-disclosedness** • Remaining secrecy and maintaining secrecy
***Undifferentiated everydayness of (being-in-the-world)***	**Endured feelings of inadequacy of *being-in-the-world-of-motherless*** • Denied *authenticity*-*of-mother-world*

### Mode of being-authenticity

#### Major lived experience theme I: experiencing an existential faith and hope

This theme emerged from the participants’ experiences of deciding to undergo IVF treatment and belonging to a world where motherhood was deemed privileged. Hope was associated not only for the desire of a child with their husband but also for a future as a mother. Faith is described as an enduring and central tenet in the lives of the women during the IVF procedure. They all expressed a sentiment of praying a lot and having faith in the Supreme Being [God or Allah] which brought hope and assisted them make *meaning* despite suffering. The sub-themes are: (i) A feeling of an ‘Existential Faith’; (ii) ‘A sense of optimism to celebrate a successful motherhood’.

#### Sub-theme: a feeling of an existential faith

The women recounted that the *essence* of faith used as a unique force helped to support their sense of self during that trying time. The process became a testament to their resilience and determination to believe in the possibility of a successful outcome.

A woman grinning in excitement as she recounts her story:

‘Religiously, it affirmed my faith in God in that I believed that I have a God who is able to do all things and that if you have God, you have everything. The more I read the Bible and sing songs of praises, the more it helped me forget my troubles and pray to God. I also said to myself that if I have life, I have everything and with God all things are possible. From that time also I read Bible a lot and was encouraged from the biblical stories of Sarah and Abraham and Hannah and prayed to God. You just replaced their names with yours and ask God to help you for a miracle to happen as He did for them.’ …. (Francisca, 32).

#### Sub-theme: a sense of optimism to celebrate a successful motherhood

The women were hopeful, and they tended to be quite optimistic about the potential for a successful conception. The participants experienced cycles of high hope and profound sorrow over repeated unsuccessful treatment cycles. They expressed feelings of high hopes when undergoing the treatment (IVF), hoping to offer them a solution to the mother world.

Juliet was hopeful and optimistic for a successful conception:

‘When I began the IVF treatment, I was hopeful. I was happy that the IVF treatment was there, and I am going to have my own child, I was happy. When I went to the hospital and the doctor said o! IVF may help you raise your own baby I was very happy since it gave me the hope that I can give birth to my own child. When I had the eggs (ovaries) too still, I was happy and optimistic because I was able to produce something, it made me feel like I am also a woman. You see when you come across successful mothers you tend to be happy because you know there is hope for you and it shall be well.’ …. (Juliet, 29).

### Mode of being-inauthenticity

#### Major lived experience theme II -facing up to the *Angst* (*Angst, Fallenness)*

The theme of Facing up to the *Angst* illustrated in this study describe how anxiety pervaded the life of the women when undergoing the IVF treatment. It was also identified from the women during the phenomenological conversation that, some medication side effects, financial constraints and uncertain outcomes of IVF treatment was not appreciated by the women. From the hermeneutic interpretive process, three main sub-themes were discovered and interpreted. The sub-themes are: (i) ‘*Angst* of uncertainty’; (ii) ‘Unpleasant nature of emotional drain’ (iii) ‘Causing huge financial losses.’

#### Sub-theme: *Angst* of uncertainty

Uncertain outcome(s) was common sub-theme shared by the women though each situation was experienced differently. The phenomenological interpretive process revealed that the outcome of the IVF treatment was never certain irrespective of every effort made. Uncertain outcome of this stressful and painful process of IVF treatment left the women feeling anxious and devastated. Nevertheless, these feelings of ‘*Angst’* were a revealing experience to overcome in *being-in-the-world* of IVF treatment when understood from a Heideggerian perspective.

A woman stated:

‘I understood that life is about taking risk and I was ready for the treatment though I had done some research which informed me that the IVF treatment was not 100% successful.’…. (Akos, 35).

The feeling of uncertainty expressed by the participants, actually turned into anxiety and stress when they were undergoing the IVF process. They also felt that the outcome of the pregnancy results was out of their control which made it more stressful. This is captured in the following narrative of one of the participants:

‘There is a lot of thought, before it would even be done for you, the processes you will even go through like the injections is painful and the screening is time consuming. After the embryo transfer has been done**,** the thinking that goes on during the first two weeks even makes it more stressful; I was uncertain either it will be successful or not? So, if it is not successful your money spent going waste or not. You pray and watch television.’…. (Mavis, 40).

However, the stressful and painful nature of this treatment should have yielded a positive, certain and absolute results, but IVF user explained the issue was different:

‘It leaves you in a lot of suspense and anxious in waiting for the outcome whether it will yield positive result was never certain.’ …. (Francisca, 32).

#### Sub-theme: unpleasant nature of emotional drain

All the women in the study agreed that the most stressful period in IVF treatment cycle is the waiting after embryo transfer to find out if the treatment has resulted in implantation and subsequent pregnancy. Several of the women stated that, the two weeks waiting period was accompanied with heightened anxiety. *Angst* of uncertainty appeared as a constant interplay between hope and doubt while waiting for pregnancy test result.

A woman described the two weeks waiting period as a roller coaster of emotions:

‘The day of embryo transfer there was mixed feelings of sadness and happiness. Why am I saying you will be sad? This is because you will be thinking about the outcome. Is it going to be successful so that my joy will be complete? You know, all the time you will be thinking about it. There is never anyone who would say she would take her mind off during that period. I for one, I was not able to sleep; I lost appetite and could not eat; it really entails thinking. So, when I come for the injection, you realise that I looked a bit pale, I lost weight, it does entail a lot of thinking. Its more than anything, there is a lot of thinking during the two weeks waiting period. I was anxious and waiting for the outcome of the results. You can’t focus on anything especially the day before the pregnancy test. The two weeks waiting period is a roller coaster of emotions.’ …. (Mavis, 40).

Another woman described the two weeks waiting period as like writing exams and wondering if you would pass or not:

‘The two weeks waiting period was full of anxiety. It was like writing an exam and wondering if you would pass or not. Whole lots of anxiety; I was anxious to know whether it was going to be positive or negative and that was not easy at all.’ …. (Lois, 40).

#### Sub-theme: causing huge financial losses

All the participants had huge financial concerns. One of the women complained of the IVF treatment causing financial loss yet the procedure was not successful. Isha was hurt, after spending such a huge sum of money but the procedure was not unsuccessful:

‘My husband works at Prudential Bank, so he took a loan from there and that was our source of finance for the procedure. In fact, we were all hurt, after spending such a huge sum of money to do it and it turns out futile, it is so painful, it’s painful. I must pay off the loan I took from the Bank.’ …. (Isha, 30).

#### Sub-theme: sense of guilt and self-blame

This lived experience sub-theme** ‘**sense of guilt and self-blame’ emerged from one of the phenomenological conversational guide with prompts: ‘How has been your experience with guilt and self-blame?’.

Akos has experienced feelings of guilt and sometimes blame herself for not preparing adequately before undergoing the IVF treatment. She recounts her experience:

‘Sometimes, I do blame myself; I thought that I could have done better before starting the process. ‘I do not blame the hospital so much because after they transferred the embryos, they have no control over it, from the little research I did, I felt it was my body that was to react with the transfer. That was how I overcame it. I didn’t have to blame somebody for my systems inability to react well with the transfer. That was the notion I got. I blamed my system for not reacting positively with it.’ …. (Akos, 35).

Mavis and Francisca also expressed similar experiences. They blamed themselves because of their lifestyle when growing up and felt guilty because of prior sexual practices some years ago and now has to face the problems.

Mavis shares her experience as this:

‘I feel guilty because my husband is interested in kids. I feel so guilty for not able to become pregnant. I also blamed myself that my body can’t respond to the IVF treatment. I for one I have done abortion on two occasions and sometimes feel guilty and blame myself that it might be as result of the abortion that is why I am experiencing this problem.’ …. (Mavis, 40).

#### Major lived experience theme III: non-disclosedness

In this study the women were challenged as to whether to disclose their IVF treatment to individuals during the phenomenological conversation. All the women in this study made a conscious effort to keep their IVF journey private, choosing not to share it with friends and family. This decision was influenced by the lack of understanding of IVF treatment in Ghana. For some, the societal norm of motherhood made it taboo to find themselves in a position where, divulging their treatment journey to anyone outside of very close family members could be challenging and stigmatised. They also feared that people will call them names and looked down upon them that was particularly an obvious reason why they decided not to disclose their IVF treatment struggle.

#### Sub-theme: remaining and maintaining secrecy

The participants grappled with the decision to keep their struggles with infertility and IVF treatment hidden, reflecting the societal and cultural pressures that led to a veil of secrecy. This subtheme highlights the internal conflicts faced by the women as they balanced the desire for support with the societal norms that often stigmatize infertility.

I asked Juliet the reason why she did not disclose the IVF treatment to anybody. She retorted:

‘Here in Ghana many don’t believe in test tube babies. People wonder if a test tube baby would live a normal life. They don’t know enough about it. When you undergo this procedure, it becomes difficult for you to discuss with friends because of lack of knowledge people have about it and the stigma attached. People usually think that individuals whose husbands have low sperm count are the ones who undergo IVF treatment.’…. (Juliet, 29).

Francisca decided to conceal the IVF treatment to avoid being the target of gossip:

‘People gossip a lot. You know this procedure people do not believe and accept it. They do not really understand it and talk too much so we decided to keep it a secret to avoid being the target of gossip and also to prevent any misunderstanding.’ …. (Francisca, 32).

### Mode of being-undifferentiatedness (everydayness of (being-in-the-world)

#### Lived experienced theme IV: endured feelings of inadequacy of *being-in-the-world-of-motherless*

This theme describes the women’s views about how living with infertility and undergoing IVF treatment has affected their everyday human *existence* of *being-in-the-world*-*of-motherless*. The women expressed feelings of disappointment and inadequacy, self-doubt, and a sense of being incomplete due to their inability to conceive and become mothers.

#### Sub-theme: denied *authenticity of mother-world*

The main sub-themes identified under Living with infertility is denied *authenticity-of-mother-world*. The women did not understand why they have been denied what every woman is expected to have without going through this kind of suffering.

Juliet was wondering if she was not a woman and did not understand why she was suffering like that as a young woman. She felt she has been denied *authenticity* of what every woman is expected to do:

‘Why am I suffering like that? I am really tired! I feel like I am not a woman. What sin have I committed? I had already heard that if you are young, it increases your chance of becoming successful, but it wasn’t working for me alone. I was wondering about the sort of problem there was with me and if someone could do something about it for me but there was no solution. The doctors don’t know what goes on during implantation. They couldn’t do anything about it. It was just like let it go and leaves the rest in the hands of God. So, we are depending on miracles to do the rest since the doctors have tried their best.’ …. (Juliet, 29).

Lois explained that she got married as a virgin and never did anything awful to deserve whatever she was going through. She narrated her story with sadness:

I was wondering if I was not a woman. Because everyone does it and it was successful. Why? Am I also not a woman just like any other women who get married and become pregnant? Why this suffering? For me not having children was not the future I had imagined since I was a virgin when I got married. The thought of ever encountering problems with conception was the least to have ever crossed my mind.’…. (Lois, 40).

## Discussion

The study revealed that the women experienced *existential* faith. The women had high hope at the beginning of the treatment trusting they would be pregnant and give birth to a healthy child and belong to the mother world. Faith helped the women endure the frustrations and difficulties experienced during the IVF treatment by making *meaning* out of their distress (*authenticity*). The women spoke of their faith or religious practices as helping them survive and cope with their loss.

Quite remarkably, the findings above also resonate with the findings of a research inquiry conducted by Mosalanejad et al. [28] and Boz and Okumus [29]. It was known from their findings that majority of the women coped with their fertility problems through coming closer to God. A study by Chan et al. [30] also found that, integrating spiritual care in psychosocial group intervention for women undergoing ART services promoted the psychological and spiritual well-being of the women diagnosed as infertile. Their result revealed that at the end of the group therapy, women described decreased levels of anxiety (*Angst*) and physical distress significantly. We agree with the suggestions of Chan et al. [30] on the need to incorporate religious and spiritual issues into current spiritual and body and mind therapies with the aim to improve spiritual care as well as the psychosocial needs of women pursuing IVF treatment.

Hope is a wish for a desired expectation [28]. Hope is one of the most important factors in IVF achievement and women begin treatment with high hopes. For all the women the struggle to remain hopeful was difficult but in spite of their disappointment, they kept the hope alive. Previous authors have also shown that there is greater amount of anxiety and distress amongst women still hoping for pregnancy [31–35].

The second concept in Heidegger's modes of existence is '*Being Inauthentic*.' In this state, *Dasein* confronts its current situation, which Heidegger describes as *fallenness* (*Verfallen*). This condition can prompt *Dasein* to conform to societal expectations and norms without critically examining them, resulting in a tendency towards conformity and a diminished sense of individuality. Heidegger underscored the significance of acknowledging this fallen condition as a crucial step in reclaiming one's *authenticity* and pursuing a more genuine existence (*Existenze*) [15, 22]. Heidegger termed the moment of disruption anxiety (*Angst*) expressed as that mood in which *Dasein*’s everyday way of *existence* in the world is characterized by anxiety and fear [19]. The overarching theme ‘Facing up to the *Angst*’ became apparent in the women’s conversation as they shared their stories of failed IVF treatment. They explained how they struggled through diverse stages of the treatment to become pregnant. Consequently, the theme** ‘**Facing up to the *Angst* reflects the ways in which anxiety clouded the women minds. Anxiety (*Angst*) is also expressed as a common response for women with fertility problems who have experienced a failed IVF treatment [29, 31, 35–38].

One of the most traumatic aspects of assisted reproductive technology (ART) is the waiting period for pregnancy results after embryo transfer. All the women in the current study reported that the greatest fear experienced in the IVF journey was the interim period post embryo transfer to determine the outcome of the procedure. This period of waiting as stated by the women causes’ great pain (*Angst*) and described the experience as the most fearful and challenging moments for their *being-in-the-world* of ART. Similar results have been reported in other studies [31–36]. The study's findings also corroborate Hammarberg, Astbury, and Baker's assertion [39] that the impact of IVF treatment is experienced during the anticipation of pregnancy results, even though the outcome is beyond the control of those undergoing the treatment.

Information gathered from the women’s narratives, indicate that the participants had concerns on the huge financial burden involved in one cycle of IVF treatment and making accessibility impossible. Many Ghanaian women are being denied access to ART services mainly due to high costs of the procedure. To make IVF services user-friendly, there is the need to reduce the cost and incorporate it in public funded facilities so that the average Ghanaian citizen can readily access the ART services.

Common to all, the women expressed guilt and self-blame about not being able to conceive. Enmeshed with guilt about the unsuccessful treatment were feelings of guilt about the cause of infertility and blaming themselves for the inability to become pregnant. Similarly, Mosalanejad et al. [28] and Durgun-Ozan and Okumuş [31] reported that the women had negative perspectives, considering all their desires and aspirations were lost and were dispirited.

Another distinctive theme that emerged from the women’s phenomenological conversation was the non-disclosedness aspect of IVF treatment. Mothers who underwent IVF treatment intentionally kept their treatment private to shield their children from potential stigma and to avoid societal misconceptions that linked IVF-conceived children with abnormalities. Some chose to conceal their IVF journey to escape gossip and unwanted attention. The secrecy surrounding infertility treatments stems from the threat they pose to the self-esteem and identity of women facing infertility, influenced by the associated stigmatization [40].

Consequently, it is explicable that the participants decided to keep their treatment from the general public domain [34]. Similar result have also been reported by Durgun-Ozan, & Okumuş [31], Ying, Wu, and Loke [35], Hammarberg [36], Hammarberg, Astbury and Baker [39], and Inhorn and Buss [41]. This theme underscores the importance of creating a supportive environment that encourages open dialogue about infertility, challenging societal stigmas, and fostering a sense of community among those facing similar challenges.

The major lived experience theme, living with infertility (*being-in-the-world-of-motherless*) includes descriptions about how the participants pursuing IVF treatment have been deprived of *authenticity* of what every woman is expected to have concerning child bearing. The women felt that the opportunity of motherhood had eluded them. Motherhood is seen as an important societal role for women in the Ghanaian culture as such all the women grieved the loss of the motherhood role. In a pronatalist country such as Ghana who advocate for bearing children and subsequent motherhood, it is essential to reproduce to ensure the continuity of family blood line or humanity as proposed by [41].

### Implications of the study for clinical practice (IVF Carers)

From the women’s narratives, it appeared that their emotional needs were not being met and were not being cared for as expected. Based on the diverse emotions experienced by participants there is a need for healthcare staff to provide emotional support to help them cope with the situation.

It is also vital for the nurses and the counsellor at the fertility clinic to be trained and fully equipped with different ways to engage the women during the two weeks waiting period and after negative pregnancy test is disclosed. The interests of incorporating psychosocial support care into standard medical programme have also been documented as helpful [33].

In addition, rendering culturally competent care remains vital in the nursing profession. Nurses and midwives must employ a high level of cultural understanding and awareness to meet the needs of the ever-revolving multicultural needs of infertility care.

The findings of this study offer valuable insights into the essential skills that healthcare providers need to acquire in order to better address the emotional needs of individuals undergoing fertility treatments. The findings guide the identification of necessary skills for effectively supporting women in the process of fertility treatment.

By gaining insights into the nuanced experiences and expectations of women facing infertility challenges, healthcare professionals can tailor their approach to provide more effective emotional support, fostering a more compassionate and patient-centered care environment.

## Limitations

This study recognizes the limited number of participants and the focused nature of the study. However, the number of participants used in this study was enough to attain thematic saturation and conclusions similar to those of other researchers [33, 34, 42]. Participants in this research sought treatment from one infertility center situated in one geographical area in Kumasi. The experiences of those women who did not seek IVF treatment for whatever reasons were not represented. The extent to which the findings reflect and resonate with other women who are infertile attending different fertility hospital remains to be determined.

## Conclusion

In conclusion, this study employed Heidegger's philosophy to illuminate the experiences of Ghanaian women facing unsuccessful invitro fertilisation (IVF) procedures. The phenomenological conversations with participants underscored that infertility and its associated treatments significantly diminish the quality of life for women. Consequently, women engaged in IVF programs require sustained emotional support across all stages of treatment.

The pervasive sense of anxiety revealed through the hermeneutic interpretive process emphasizes the profound impact of IVF on women's quality of life. When confronted with the failure of IVF treatment, participants expressed a poignant belief that the opportunity to become a mother had eluded them. This emotional toll reflects the substantial disappointment and distress accompanying unsuccessful fertility interventions.

Notably, there is a pressing need to establish realistic patient expectations regarding IVF success rates at the outset of treatment. The study further illuminated that, women facing the prospect of future infertility, experienced distress and a sense of loss. The risk of societal and cultural pressures was evident as participants grappled with the decision to conceal their struggles with infertility and IVF treatments, highlighting the prevalence of a veil of secrecy.

The study's findings also shed light on the potential negative repercussions of idealistic reassurances regarding IVF success. While optimism is important, addressing these idealizations is crucial to aiding women in adapting to negative outcomes. Participants revealed grappling with feelings of disappointment, inadequacy, self-doubt, and a sense of incompleteness due to their inability to conceive and fulfill societal expectations of motherhood.

In essence, this study underscores the multifaceted challenges faced by women undergoing IVF in Ghana, emphasizing the necessity for ongoing emotional support, transparent communication about success rates, financial ramificatication and the importance of dismantling societal taboos surrounding infertility and IVF treatment. By acknowledging and addressing these complex emotional and cultural dynamics, healthcare providers can better support women navigating the intricate journey of infertility and fertility treatments.

## Data Availability

A complete document of this study and its results can be found at the Library of the University of Cape Town, South Africa. The transcripts from which this study was written are also available on request from the corresponding author.

## Abbreviations

ART: Assisted Reproductive Technology
IVF: Invitro Fertilisation
CHRPE: Committee on Human Research, publications and Ethics
HREC: Health Science Research Ethics Committee
KNUST: Kwame Nkrumah University of Science and Technology

## Footnotes

1 ‘An ‘entity (*entities)’* or a being (*ein Seiendes*) is anything that in any sense is as an object or things ‘(Gorner, 2007, p. 15).

2 Something capable of being apprehended by the mind alone.
